# Intraparenchymal Anaplastic Meningioma With Isolated Motor Deficits

**DOI:** 10.7759/cureus.89822

**Published:** 2025-08-11

**Authors:** Ayushi Garg, Nour Aldaoud, Wajahat Khan, Zain I Kulairi

**Affiliations:** 1 Internal Medicine, Wayne State University School of Medicine/Henry Ford Rochester Hospital, Rochester Hills, USA; 2 Medical Oncology, Henry Ford Rochester Hospital, Rochester Hills, USA

**Keywords:** anaplastic meningioma, brain tumor, craniotomy, focal neurological deficits, frontal lobe mass, high-grade meningioma, histopathology, intra-axial, who grade iii

## Abstract

This case report describes a 62-year-old female with a history of gastroesophageal reflux disease and irritable bowel syndrome who presented with progressively worsening left-sided weakness and gait instability of two weeks’ duration. A CT of the head without contrast revealed a 3.7 × 3.6 cm right frontal lobe mass with vasogenic edema and significant midline shift. A subsequent brain MRI with and without contrast demonstrated a 5.8 × 4.6 × 4.2 cm intra-axial, aggressive-appearing mass centered posteriorly within the right frontotemporal lobe, with extensive surrounding edema and local mass effect. The patient underwent surgical resection via craniotomy, with subsequent resolution of focal deficits. Histopathology confirmed an anaplastic meningioma, WHO Grade III, despite the fact that meningiomas are typically extra-axial tumors. This case underscores that high-grade meningiomas, although rare, can present in unexpected ways and at unpredictable sites, creating diagnostic challenges. It also emphasizes the importance of timely neurosurgical intervention to improve the chances of recovery.

## Introduction

Meningiomas are typically extra-axial tumors originating from arachnoid cap cells, accounting for approximately 13-26% of all intracranial tumors [1]. According to WHO, they are classified into three grades: benign (Grade I), atypical (Grade II), and anaplastic or malignant (Grade III) [2]. While most meningiomas are benign, a small percentage (1.0-2.8%) are malignant, with metastasis being extremely rare (0.1%) [1,3].

Anaplastic meningiomas (WHO Grade III) are rare and aggressive, accounting for 1-2% of all cases [4]. They grow rapidly, invade the brain, and have a worse prognosis compared with lower-grade tumors. While most meningiomas are located in the extra-axial space, intraparenchymal meningiomas are exceptionally rare, presenting a diagnostic challenge. Timely surgical resection is important to reduce the risks of metastasis, morbidity, and mortality. Studies have shown the significance of gross total resection (GTR) in improving survival outcomes, with one analysis of 755 patients reporting a five-year overall survival (OS) rate of 41.4% for anaplastic meningiomas [5].

This report describes an unusual case of anaplastic meningioma presenting within the intra-axial space and with isolated motor deficits, underscoring the need for thorough neurological evaluation to ensure timely surgical intervention in similar presentations. It also highlights the necessity of histopathology in guiding further treatment plans by determining the specific diagnosis and aggressiveness of brain tumors, especially when they occur at rare sites, as in our case of intraparenchymal anaplastic meningioma.

## Case presentation

We present a case of a 62-year-old female with a past medical history significant for gastroesophageal reflux disease and irritable bowel syndrome who presented to the hospital with progressive left-sided weakness and gait instability of two weeks’ duration. The patient denied new-onset headaches, visual changes, speech difficulties, facial droop, sensory disturbances, chest pain, shortness of breath, or any other significant acute complaints. She had no personal history of malignancy, cardiovascular disease, or stroke. Family history was notable for breast cancer in her maternal aunt. Physical examination revealed mild left lower extremity weakness.

Routine tests, including a complete blood count, renal and liver function tests, and an electrocardiogram, were all within normal limits. On examination, left hip flexion and knee extension were Medical Research Council grade 4/5; dorsiflexion was 3/5; all other muscle groups were intact.

**Figure 1:**
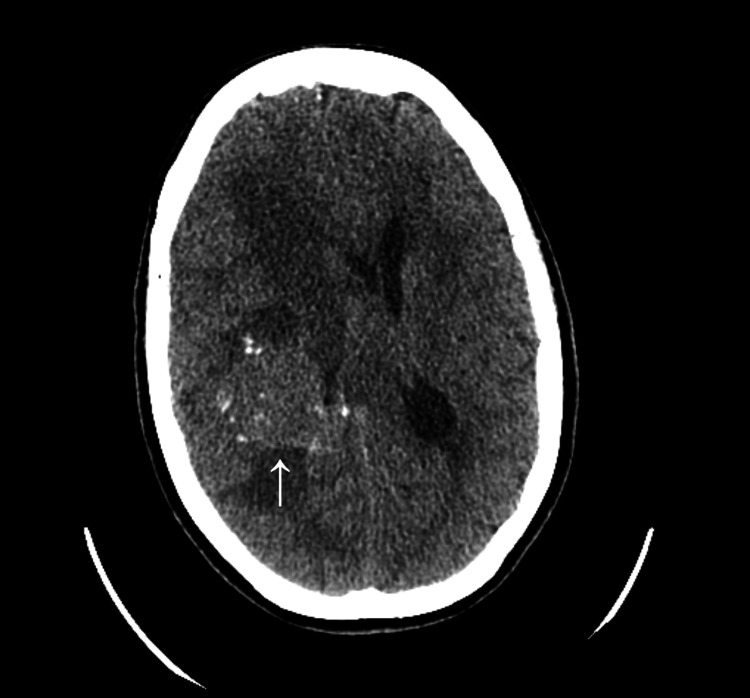
CT scan of the head without contrast showing a hyperattenuating, partially calcified mass (white arrow) measuring approximately 3.7 × 3.6 cm, with surrounding vasogenic edema involving the frontotemporal region.

A non-contrast CT scan of the brain revealed a 3.7 × 3.6 cm mass in the right frontal lobe, with surrounding swelling and a 1.1 cm midline shift toward the left (Figure 1). The initial differential diagnosis included a primary brain tumor or metastatic cancer.

**Figure 2:**
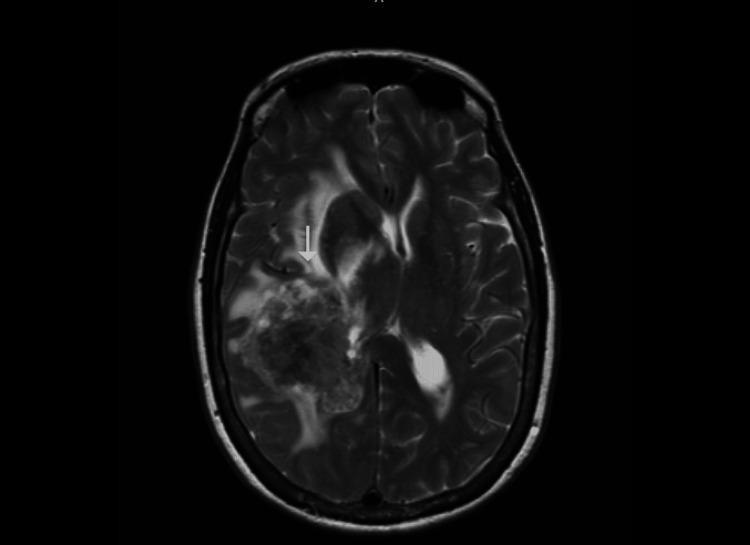
MRI of the brain with and without contrast (T2 sequence) showing an approximately 5.8 cm intra-axial heterogeneous mass (white arrow) in the right frontotemporal lobe, with extensive surrounding edema and local mass effect on the ventricles and midline shift.

MRI with and without contrast confirmed a 5.8 × 4.6 × 4.2 cm intra-axial, aggressive-appearing mass centered in the posterior right frontotemporal lobe, with extensive surrounding edema and local mass effect (Figure 2). A CT scan of the chest, abdomen, and pelvis was then performed to rule out other primary malignancies or metastases, which yielded negative results.

The patient was admitted and started on dexamethasone 4 mg every six hours to manage cerebral edema, along with levetiracetam for seizure prophylaxis.

After thorough discussions among the neurosurgery team, the oncology team, the patient, and her family, the decision was made to proceed with surgical tumor removal. Under general anesthesia, the head was secured in a Mayfield clamp in a three-quarter lateral position. Intraoperative neuronavigation with cortical mapping guided a horseshoe-shaped frontotemporal craniotomy to avoid the eloquent cortex. The dura was opened in a cruciate fashion, and no dural attachment of the tumor was identified.

A well-demarcated, dense, fibrous lesion was accessed via a small corticotomy, debulked under the operative microscope, and carefully dissected away from the surrounding brain and choroid plexus to achieve GTR. Hemostasis was maintained throughout using bipolar cautery and absorbable hemostatic agents. Motor-evoked potential monitoring was employed to preserve function. No intraoperative photographs were available.

**Figure 3:**
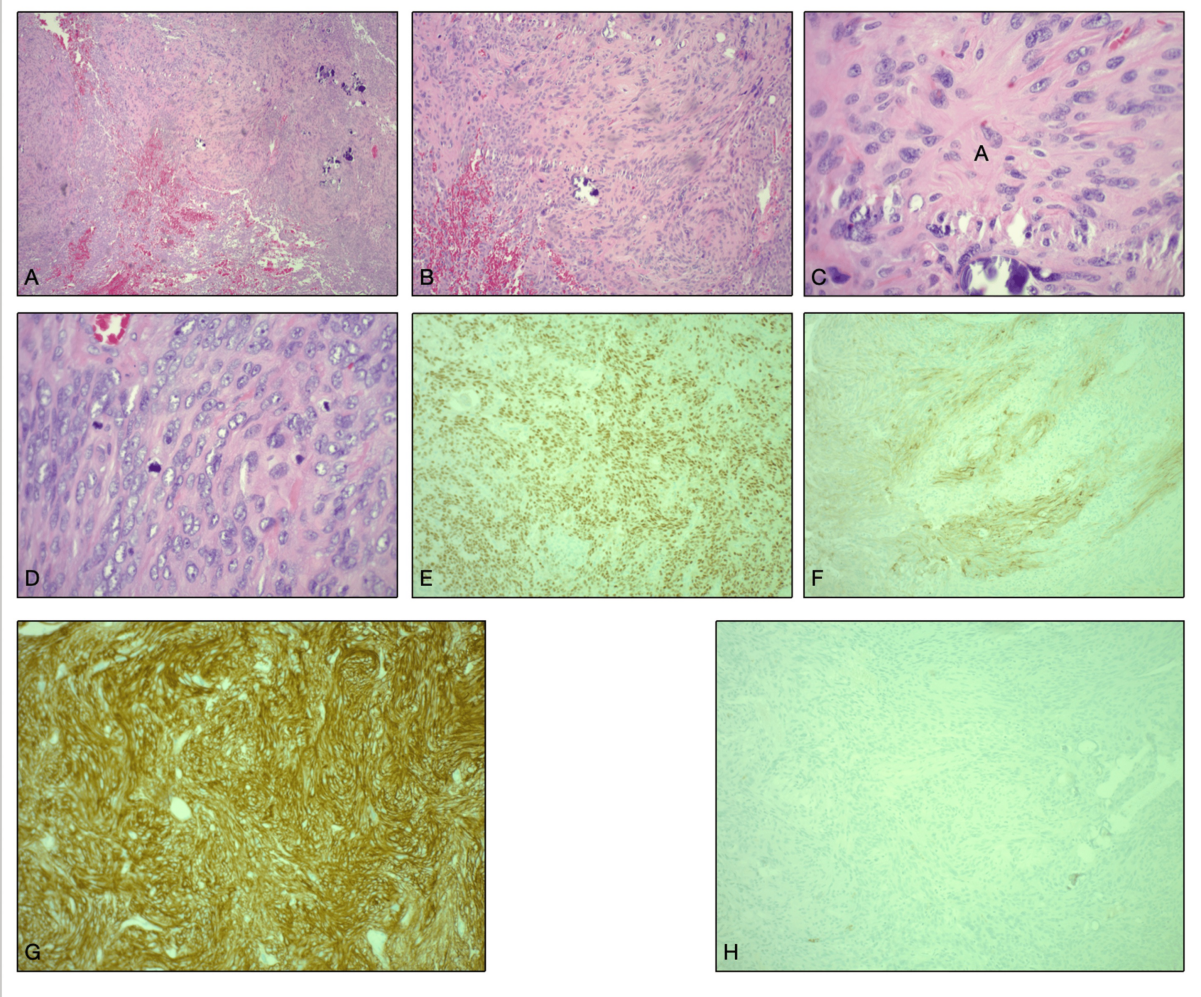
Histopathological analysis of the mass: (A) Low magnification, 40×. (B) Medium magnification, 100×. (C) High magnification, 400×. (D) High magnification showing 4 mitoses in one field. (E) Positive immunohistochemistry for epithelial membrane antigen, characteristic of meningioma. (F) Positive immunohistochemistry for progesterone receptor, nuclear positivity, characteristic of meningioma. (G) Positive immunohistochemistry for somatostatin receptor, membrane positivity, which is sensitive but not specific for meningioma. (H) Negative immunohistochemistry for glial fibrillary acidic protein, characteristic of meningioma.

Histopathological analysis identified the mass as an anaplastic meningioma, WHO Grade III, characterized by high cellularity, pleomorphism, and a high mitotic index (Figure 3).

Histopathological analysis confirmed the diagnosis of WHO Grade III anaplastic meningioma. The tumor showed high cellularity, pleomorphism, and a high mitotic index. Immunohistochemistry was positive for epithelial membrane antigen, progesterone receptor, and somatostatin receptor 2A, while glial fibrillary acidic protein was negative, confirming the tumor’s non-glial origin (Figure 3). These findings emphasized the aggressive nature of the tumor.

Postoperatively, the patient was monitored in the ICU, and her focal lower extremity motor strength gradually improved from 4/5 to 5/5. On postoperative day 4, she was discharged on dexamethasone and levetiracetam, with outpatient follow-up scheduled with neurosurgery and oncology. Following pathological confirmation of Grade III disease, the patient was referred for adjuvant radiotherapy.

At her three-month follow-up, a brain MRI showed no residual tumor, and the previously noted midline shift had resolved. A tiny focus of enhancement along the posterior margin of the resection cavity was noted, likely representing post-surgical granulation or reactive changes rather than residual tumor. At six months, a repeat MRI revealed stable post-surgical changes with no new or progressive intracranial enhancement. The previously observed focus of enhancement had resolved, further supporting its benign post-surgical nature.

These findings, along with the patient’s stable neurological status, suggest a positive postoperative course. While her neurological condition remained stable and recovery was progressing well, she continued to be closely monitored due to the high risk of recurrence with Grade III anaplastic meningiomas.

## Discussion

While most meningiomas are benign and treatable through surgical resection, atypical (Grade II) and anaplastic (Grade III) meningiomas have a higher likelihood of recurrence [2]. Anaplastic meningiomas may be high-grade from the outset or may develop through malignant transformation from lower-grade tumors [6].

Anaplastic meningiomas are typically extra-axial, located outside the brain parenchyma, making their diagnosis and management relatively straightforward. However, this case report illustrates the rare occurrence of an intra-axial anaplastic meningioma, which complicated the radiological and histopathological assessment. The tumor’s location within the brain parenchyma, rather than the expected extra-axial space, led to initial diagnostic uncertainty, underscoring the critical role of histopathologic and radiologic correlation for timely intervention.

Imaging techniques such as brain CT and MRI are essential in assessing the size, edema, and mass effect of brain tumors, with postoperative histopathological analysis confirming the diagnosis and providing valuable insights into the tumor’s potential aggressive biological behavior. In this case, the patient was diagnosed with a high-grade intra-axial anaplastic meningioma. Because meningiomas are usually extra-axial tumors, this case represents an atypical intra-axial site, posing a challenging radiological diagnosis. Corticosteroids were used to reduce cerebral edema, and anticonvulsants were administered for seizure prophylaxis. Achieving GTR, which is critical in treating anaplastic meningiomas, provided the best chance for short-term recovery.

While GTR is essential for immediate treatment success, anaplastic meningiomas are known for their high recurrence rates. The use of adjuvant radiotherapy remains debated, with some studies suggesting it may reduce recurrence risk [7]. Consequently, the patient was referred for adjuvant radiotherapy following discharge. Follow-up MRIs at three and six months showed no recurrence. These favorable results underscore the vital role of prompt surgical intervention and highlight the importance of tailored postoperative care and monitoring. However, the long-term prognosis remains poor due to the high likelihood of local recurrence, even after GTR.

In addition to complete resection, adjuvant radiation therapy (RT) is commonly used, especially in patients with incompletely resected anaplastic meningiomas, to improve local disease control [8]. Research has demonstrated that RT can benefit patients with malignant meningiomas and may improve OS and progression-free survival in both primary and recurrent anaplastic meningioma cases [9-11]. Dziuk et al. reported that combining surgery with RT resulted in better five-year disease-free survival compared to surgery alone [12].

Recurrent anaplastic meningioma exhibits more aggressive biological behavior despite surgical interventions and often requires chemotherapy, especially after failure of surgery and radiation. However, chemotherapeutic agents such as hydroxyurea, bevacizumab, temozolomide, and irinotecan have shown limited efficacy in reducing recurrence in high-grade meningiomas [13-16]. Therefore, further treatment options need exploration to improve survival in such cases.

This report emphasizes the essential role of pathology in both diagnosis and treatment guidance. Histopathological analysis is crucial for confirming tumor grade and understanding biological behavior, providing valuable insights for immediate care and long-term prognosis. In this case, pathological examination revealed the tumor to be a WHO Grade III anaplastic meningioma, characterized by high cellularity, pleomorphism, and a high mitotic index, despite the tumor’s unusual intra-axial location. These findings not only confirmed the diagnosis but also determined the tumor’s aggressive nature, further guiding the treatment approach and follow-up strategy.

## Conclusions

Our case of anaplastic meningioma highlights the concern for malignant brain tumors, particularly when located in unexpected brain regions in patients presenting with focal neurological symptoms. Early diagnosis, prompt imaging, and timely surgical intervention are critical for managing anaplastic meningiomas and improving outcomes. Histopathological analysis provided essential insights into the tumor’s grade and behavior, especially considering the atypical intra-axial location, directly guiding treatment decisions and follow-up care. Despite successful surgical resection and stable postoperative imaging, the high recurrence rate associated with these tumors necessitates vigilant long-term surveillance.
